# Stability and variability in income position over time: exploring their role in self-rated health in Swedish survey data

**DOI:** 10.1186/1471-2458-14-1300

**Published:** 2014-12-18

**Authors:** Alexander Miething, Monica Åberg Yngwe

**Affiliations:** CHESS | Centre for Health Equity Studies / Stockholm University, SE-106 91 Stockholm, Sweden

**Keywords:** Social inequality in health, Income change, Income position, Self-rated health, Sweden

## Abstract

**Background:**

The adverse relationship between income and health is well documented, but less is known of how income trajectories, i.e. downward or upward trends in income, determine health. We therefore link longitudinal income information to cross-sectional data on self-rated health and conduct logistic regression models in order to investigate income trends over time and their relationship with health.

**Methods:**

The data, with the exception of income information, are derived from the Swedish Level-of-Living Survey 2000. The information on income was drawn from the income register covering the period 1995 to 2000. We used an age-restricted sample of those 30–64 years of age, and generated a series of models accounting for average income position, lagged income position, income decrease and increase, and annual periods in specific income positions. The analysis was conducted for men and women separately.

**Results:**

Apart from the overall association between income and health, we found a similar pattern when including average and lagged income in the model. The analysis of length of time in a specific income position showed substantial sex differences in poor health. Income decrease was more strongly associated with men’s poor health, whereas income increase revealed only weak associations with self-rated health.

**Conclusions:**

It was shown that income changes and the time dimension of income are important for self-rated health. Self-rated health responds to decreases in absolute income and lowered rank position in the income distribution to a greater extent than to income gains over time. Lagged lower income position and its associations with health suggest that socio-economic disadvantages accumulate over time.

## Background

Extensive research has been dedicated to demonstrating the adverse health consequences of low income position; however, much less attention has been paid to income dynamics and lagged effects of income [1]. The primary reason why income dynamics (i.e. downward and upward trends in income) are relatively unexplored is the common application of cross-sectional data, which are naturally limited when dealing with trends over time. Cross-sectional data capture only current income, whereas previous income histories are omitted. Disregarding income dynamics neglects the fact that income is by no means stable over time, as cross-sectional approaches might imply [2]. In monitoring the time dimension of income, longitudinal approaches have several benefits over cross-sectional analysis: Reversed causality and health selection can be reduced more efficiently; dynamics and income variation over time can be captured; and repeated measures of income are less error-prone than occasional income from a single year.

Research on income inequalities in health has mainly focused on the income-specific mechanisms involved in the relationship, such as absolute income mainly drawing on material resources or relative income addressing non-material aspects and social comparisons [3–5]. In regard of income, relatively few approaches to date have accounted for income variability with respect to health. A longitudinal approach by Benzeval and Judge [6] has explored income changes and their role in various health measures, combining income series with cross-sectional information on health. Using a set of self-assessed indicators, the study revealed that average income over a five-year period had the strongest effects on health, suggesting that the cross-sectional approach underestimates the (true) relationship between income and health. Persistent poverty revealed the highest risk for poor subjective health; scores based on average income were substantially higher than those from single annual periods in poverty. Initial income level appeared to be more significant than changes in income. Falling income revealed greater importance for health than did income increases [6]. A study by McDonough et al. [7] based on US survey data found increased mortality rates for middle-class individuals who had experienced substantial income losses. For low-income earners, a sharp income decline did not exceed the mortality risk they already had. For high-income earners, no significant increases in mortality risk were found [7].

Earlier studies have almost consistently shown that increasing income is more beneficial for health than falling income [8]. The extent to which income increase influences health is covered only weakly in earlier studies. In comparisons of stable income with increased and decreased income, the health implications are by far less coherent. In a longitudinal survey from New Zealand, Gunasekara et al. [9] found a very weak increase in self-rated health for those with noteworthy income increase of more than NZ$10,000. Only those with low income at baseline and income increase had notable health benefits. Empirical evidence regarding the role of lagged income effects has mainly been provided on the basis of community-level analysis [1]. Investigating state-level inequalities and their lagged effects in US federal states, Blakely et al. could show that income inequality up to 15 years beforehand demonstrated significant associations with self-rated health [10].

Previous literature is not particularly clear about the causal mechanisms involved in the complex of income change and health. However, earlier research has revealed that the discontinuation of specific aspects in people’s life (like the disruption of access to resources) and certain events can trigger health problems and deteriorate well-being. In this respect, falling income evokes a frustrating experience that might initially trigger stress reactions. In the long run, a fall in income is accompanied by material shortages and less purchasing power, the loss of social integration and feelings of deprivation, which might also lead to health problems such as distress [4, 11].

The health implications of income increase appear to be ambiguous. Looking at previous studies, there is no consistent proof that income increase entails health improvements. Obviously, higher income increases the ability to consume, but it must be questioned to what extent this can be converted into better health. Depending on individual priorities, additional income can be invested in healthy food and lifestyle or health care, but also in consumption goods and hazardous activities, which in turn might deteriorate one’s health.

A further question concerns the role of contextual conditions, like welfare provisions, which are able to mitigate the consequences of low income, income loss, unemployment, poverty and related economic hardships. Regarding low income position and poverty, it has often been argued that these conditions are less severe in the Swedish context since Sweden has a relatively generous welfare system that provides comprehensive social security, parachuting basically everyone in society.

### Aims of the study

The present study hypothesizes that the individual’s current income situation is crucial for health, and further that income changes *per se* contribute to social inequalities in health. The specific aim of this paper then is to explore short and longer-term consequences of individual income position on health in Sweden, and further, to account for income variations over a six-year period by combining longitudinal income information with self-rated health from a cross-sectional survey. In a somewhat explorative approach, different modeling strategies are used in order to identify income developments in terms of both changes and stability over time. The proposed analysis distinguishes between health effects due to income level and income change by adding the respective covariates that distinguish the two dimensions. Of particular interest is the extent to which income changes affect people’s health in their respective income position; whether income changes have similar health consequences for those in low and high income groups. Further, enduring low-income position and its health consequences will be tested.

## Methods

The data for this study were derived from the Swedish Level-of-Living Survey (LNU), using the latest available cross-section with data from the year 2000 comprising a total sample of 5,142 individuals [12]. The cross-sectional information is linked to Swedish income registers, which allowed the assessment of income information for the 1990s. Access and permission to use data was given by the LNU Research Group at the Swedish Institute for Social Research. For all presented calculations, individual disposable income was used (i.e. income after taxation and welfare transfers). In order to rule out the strong income fluctuations in the aftermath of the deep economic recession in Sweden in the early 1990s, we only considered income developments between 1995 and 2000.

With respect to the quasi cross-sectional data and the categorical nature of the variables of interest, the analysis was conducted using logistic regression. In one of the analytical models, OLS regression was additionally applied. All calculations were performed in Stata 11. An age-restricted sample of persons between 30 and 64 years was used to capture those mainly receiving working income. The income pooled sample size thus covered about 3,377 individuals. All models were adjusted for age of years of the respondents.

### Self-rated health status

The health measure used in this study was self-rated health (SRH). The reliability and applicability of this measure has been frequently discussed [13, 14]. The question for SRH is “How do you judge your own general health?”, with the three response options “good”, “bad” and “something in between”. In order to apply logistic regression, the responses were recoded into a binary variable. The responses “bad” and “something in between” were aggregated into “poor” SRH.

### Educational degree

Like SRH, information on the individual’s educational attainment was derived from the LNU cross-section. The level of education an individual attains can be regarded as reliable indicator of later socio-economic position. Because educational degrees remain relatively stable throughout the life course, the use of education as a covariate may reduce the effects of social selection and reverse causality between income and health in the presented analysis. The education variable reflects highest attainment and was categorized into “primary/no degree”, “secondary” and “post-secondary” levels.

### Income change and stability

The modeling of income change and income stability was based on the pooled income information we obtained for the years 1995 to 2000. The income quintiles were calculated on the basis of the mean income over time and considered the income distribution of the total sample. Because the using sample was age-restricted and stratified by sex, the initially coded income quintiles resulted in unevenly distributed income groups. In addition, a set of income-based covariates was generated to assess income changes over time, to explore whether changes in income position or absolute income were important.

1. Length of time below median income
  To measure enduring low income, a variable was created that considers the number of years in the income position below the income median. The median was calculated separately for each calendar year. This covariate consists of seven categories, ranging from “never” (0 years) below median up to a maximum of six years with below-median income.

(2) Changes in the rank position in the income distribution
  Changes in income position were analyzed by considering the variations of income position between 1995 and 2000. Relative income position was assessed by creating income percentiles, which gave 100 income subgroups of equal size. The calculation of percentiles was based on the total sample for which income information was available. The income percentiles for the period 1995 to 2000 were then used to trace changes in the rank position in the income distribution over time. First, all changes were tracked by calculating the difference in percentiles of income between the current and the preceding income year (t0 - t1; t1 - t2; etc.). In the next step, a variable was created which kept all substantial changes and non-changes. This variable consisted of four distinct categories and considered all changes with more than ten percentiles from one year to another. The reference category included all those in the sample with a stable income (i.e. no changes larger or smaller than ten percentiles compared to the preceding income position). In the second category, all those without income increase were included (i.e. those who experienced periods with stable income in combination with income decrease). The third category captured those without income decrease (i.e. with stable income in combination with income increase). The fourth category included all others (i.e. those without a particular trend during the considered period). This group, labeled as “inconsistent”, included all subjects who had not only periods of income increase and decrease but also stable income periods.

(3) Relative changes in absolute income
  In order to assess the relative changes in the nominal amount of income, we explored how a relative change with amplitudes of more than ten percent (+/- 10%) in absolute income affected the health outcome. We chose this approach to test the extent to which a substantial income change in relation to the income level would affect the health outcome. In this approach we took into account that an income change – particularly loss of income – has different meaning and consequences for those in higher or lower income positions likely with less severe consequences for those with high income.
  Similar to the approach in changes in the rank position, four distinct groups with income developments over time were specified. The reference group included all those with stable income. The second category captured those without income increase. The third category accounts for individuals without income decrease, and the fourth, labeled as “inconsistent”, subsumed all others with downward and upward income changes during the observation period.

(4) Regression based slopes
  Finally, income trends over time were assessed through the slopes of linear regression. Regression slopes were calculated for each individual capturing the income developments between 1995 and 2000. The obtained b-coefficient of the slope then was recoded into a five-fold ordinal variable, reflecting the following categories: “strong decrease”, “weak decrease”, “stable income”, “weak increase” and “strong increase”.
  The slope extrapolates income development and reflects the individual income trends in a very crude way. A strong increase then corresponds to a gain of more than 10,000 SEK, weak increase between 10,000 and 1,000 SEK. Stable income was defined as the range between 1,000 and -1,000 SEK. Weak and strong decrease mirrored the threshold for income increase. Analogue to income increase, income decreases were defined as “strong” when income dropped with more than 10,000 SEK and as “weak” for losses between 1,000 and 10,000 SEK over time.

## Results

An overview of the distribution of the variables, the prevalence of poor health and levels of absolute disposable mean income during the observation period are shown in Table 1.

**Table 1:** **Variables and distribution of frequencies**

Variables	Men			Women		
	Number of cases	Proportion (%) of poor SRH	Absolute disp. mean income 1995 to 2000, in thousands of SEK	Number of cases	Proportion (%) of poor SRH	Absolute disp. mean income 1995 to 2000, in thousands of SEK
**Highest achieved school degree**						
Post-secondary	255	16.5	224	229	17.9	157
Secondary	1,105	22.9	169	1,141	27.2	133
Primary/incomplete	341	33.4	143	306	39.9	109
**Disposable income (pooled income 1995 to 2000)**						
1st quintile (highest)	637	17.8	248	280	17.1	211
2nd quintile	468	25.7	154	399	27.3	151
3rd quintile	321	29.3	125	446	27.6	124
4th quintile	176	31.8	97	375	34.1	98
5th quintile (lowest)	99	26.3	53	176	36.9	50
**Time in income position below median**						
0 (above median during entire observation)	983	19.8	210	550	22.0	180
1 year	140	27.9	170	144	27.1	146
2 years	119	31.1	132	139	22.6	131
3 years	105	25.7	128	120	25.0	119
4 years	83	35.0	116	114	29.8	113
5 years	88	30.7	98	148	38.5	105
6 years	183	30.0	80	461	35.1	85
**Income change: more/less than 10 percentiles in income rank position**						
Stable	711	18.3	206	590	27.6	133
Stable/downward	268	34.3	151	282	33.7	129
Stable/upward	234	25.2	155	273	20.2	132
Inconsistent	488	26.2	142	531	30.1	130
**Income change: more/less than 10% of absolute income**						
Stable	745	24.3	172	737	27.8	124
Stable/downward	78	44.9	150	118	38.1	122
Stable/upward	596	20.6	180	571	22.7	143
Inconsistent	282	24.8	161	250	37.2	132
**Slopes (based on disposable income 1995 to 2000)**						
Strong decrease	69	35.8	180	50	24.0	141
Weak decrease	212	37.3	143	240	34.6	125
Stable income	136	30.1	137	169	39.6	106
Weak increase	785	22.8	155	824	27.7	128
Strong increase	499	17.0	220	393	21.1	153
Total	1,701	24.0	172	1,676	28.2	131

### Model 1: Length in time below median

In Model 1 (Table 2) we explored lower income position across the period 1995 to 2000 using the pooled income information. The reference category included individuals whose disposable income did not fall below the median in any of the calendar years during the entire observation period (1995 to 2000). Contrast categories were defined by number of calendar years in income position below median. The median threshold was chosen since the analysis with alternative cut-points produced similar magnitudes, but also showed rather inconsistencies in patterns over time. Therefore, only findings below median income were shown. The analysis of the male sample indicates that already a single episode in low income position increases odds ratios (OR) for poor SRH compared to those in the reference group. After three years with low income the estimates increased once more, and remained relatively stable at a high level. For women, five and six years in income position below median increases the association with poor SRH significantly.

**Table 2:** **Logistic regression for poor SRH and income position below median**

Model 1	Men		Women	
	OR	CI (95%)	OR	CI (95%)
Age	1.05	(1.04 - 1.07)	1.03	(1.02 - 1.04)
**Highest achieved school degree**				
Post-secondary	1		1	
Secondary	1.47	(1.01 - 2.12)	1.45	(1.00 - 2.10)
Primary/incomplete	1.76	(1.16 - 2.67)	2.02	(1.31 - 3.12)
**Time in income position below median**				
0 (above median during entire observation)	1		1	
1 year	1.64	(1.08 - 2.49)	1.39	(0.91 - 2.14)
2 years	2.26	(1.46 - 3.51)	1.04	(0.66 - 1.64)
3 years	1.58	(0.98 - 2.56)	1.25	(0.78 - 1.99)
4 years	2.47	(1.50 - 4.05)	1.55	(0.98 - 2.45)
5 years	2.02	(1.23 - 3.33)	2.15	(1.45 - 3.20)
6 years	1.90	(1.31 - 2.75)	1.67	(1.25 - 2.23)
Observations	1,701		1,676	

*Notes:* Odds ratios. All covariates mutually adjusted.

### Model 2: Relative income position

Model 2 (Tables 3 and 4) shows the associations between relative income level based on the mean income from 1995 to 2000 and SRH. The income-health gradient is clearly present for men and women, although the lowest income quintile in males does not correspond to the highest risk of poor SRH. When adding the covariates for income change in models 3 through 5, the income estimates somewhat attenuate.

**Table 3:** **Logistic regression for poor SRH, relative level of income and changes in income over time**

Men	Model 2		Model 3		Model 4		Model 5	
	OR	CI (95%)	OR	CI (95%)	OR	CI (95%)	OR	CI (95%)
Age	1.05	(1.04 - 1.07)	1.06	(1.04 - 1.07)	1.05	(1.04 - 1.07)	1.05	(1.04 - 1.06)
**Highest achieved school degree**								
Post-secondary	1		1		1		1	
Secondary	1.35	(0.93 - 1.96)	1.30	(0.90 - 1.90)	1.36	(0.94 - 1.98)	1.35	(0.93 - 1.98)
Primary/incomplete	1.53	(1.00 - 2.35)	1.51	(0.98 - 2.32)	1.53	(1.00 - 2.36)	1.54	(1.00 - 2.38)
**Disposable income (pooled income 1995 to 2000)**								
1st quintile (highest)	1		1		1		1	
2nd quintile	1.63	(1.20 - 2.21)	1.37	(1.00 - 1.89)	1.64	(1.20 - 2.22)	1.61	(1.17 - 2.20)
3rd quintile	2.19	(1.57 - 3.05)	1.76	(1.24 - 2.49)	2.16	(1.55 - 3.03)	2.06	(1.46 - 2.90)
4th quintile	2.62	(1.76 - 3.90)	2.15	(1.43 - 3.24)	2.48	(1.66 - 3.72)	2.38	(1.58 - 3.58)
5th quintile (lowest)	1.91	(1.14 - 3.20)	1.60	(0.95 - 2.70)	1.85	(1.09 - 3.14)	1.69	(1.00 - 2.85)
**Income change: more/less than 10 percentiles in income rank position**								
Stable			1					
Stable/downward			2.02	(1.44 - 2.84)				
Stable/upward			1.81	(1.24 - 2.65)				
Inconsistent			1.61	(1.19 - 2.18)				
**Income change: more/less than 10% of absolute income**								
Stable					1			
Stable/downward					2.02	(1.22 - 3.33)		
Stable/upward					0.94	(0.71 - 1.24)		
Inconsistent					0.82	(0.58 - 1.15)		
**Slopes of income over time (based on disposable income 1995 to 2000)**								
Strong decrease							1.48	(0.78 - 2.79)
Weak decrease							1.42	(0.88 - 2.29)
Stable income							1	
Weak increase							0.82	(0.54 - 1.24)
Strong increase							0.82	(0.52 - 1.31)
*Linear trend of categories of slope*							0.83	(0.75 - 0.92)
Observations	1,701		1,701		1,701		1,701	

*Notes:* Odds ratios. All covariates mutually adjusted.

**Table 4:** **Logistic regression for poor SRH, relative level of income and changes in income over time**

Women	Model 2		Model 3		Model 4		Model 5	
	OR	CI (95%)	OR	CI (95%)	OR	CI (95%)	OR	CI (95%)
Age	1.03	(1.02 - 1.04)	1.03	(1.02 - 1.04)	1.03	(1.01 - 1.04)	1.03	(1.01 - 1.04)
**Highest achieved school degree**								
Post-secondary	1		1		1		1	
Secondary	1.43	(0.99 - 2.08)	1.44	(0.99 - 2.10)	1.43	(0.99 - 2.08)	1.44	(0.99 - 2.09)
Primary/incomplete	1.94	(1.26 - 2.99)	1.99	(1.29 - 3.08)	1.87	(1.21 - 2.89)	1.90	(1.23 - 2.93)
**Disposable income (pooled income 1995 to 2000)**								
1st quintile (highest)	1		1		1		1	
2nd quintile	1.77	(1.21 - 2.60)	1.72	(1.16 - 2.55)	1.78	(1.21 - 2.63)	1.77	(1.19 - 2.61)
3rd quintile	1.82	(1.24 - 2.69)	1.64	(1.11 - 2.42)	1.72	(1.17 - 2.53)	1.67	(1.12 - 2.47)
4th quintile	2.35	(1.59 - 3.46)	2.24	(1.52 - 3.32)	2.32	(1.57 - 3.43)	2.19	(1.47 - 3.27)
5th quintile (lowest)	2.37	(1.51 - 3.71)	2.39	(1.52 - 3.76)	2.24	(1.41 - 3.55)	2.15	(1.36 - 3.40)
**Income change: more/less than 10 percentiles in income rank position**								
Stable			1					
Stable/downward			1.41	(1.03 - 1.94)				
Stable/upward			0.78	(0.55 - 1.12)				
Inconsistent			1.32	(1.00 - 1.75)				
**Income change: more/less than 10% of absolute income**								
Stable					1			
Stable/downward					1.50	(0.99 - 2.28)		
Stable/upward					0.81	(0.62 - 1.06)		
Inconsistent					1.34	(0.98 - 1.85)		
**Slopes of income over time (based on disposable income 1995 to 2000)**								
Strong decrease							0.59	(0.28 - 1.23)
Weak decrease							0.91	(0.60 - 1.39)
Stable income							1	
Weak increase							0.67	(0.47 - 0.96)
Strong increase							0.61	(0.41 - 0.93)
*Linear trend of categories of slope*							*0.90*	*(0.81 - 0.99)*
Observations	1,676		1,676		1,676		1,676	

*Notes:* Odds ratios. All covariates mutually adjusted.

### Model 3: Changes in rank position in income distribution over time

In Model 3 the relative change in income position was explored. The analysis reveals that a substantial downward change in relative income position increases the risk for poor health – stronger for men than for women. Income loss combined with stable income showed increased ORs for both men (OR 2.02, CI 95% 1.44-2.84) and women (OR 1.41, CI 95% 1.03-1.94) compared to the reference category. The gender differences are even more pronounced for income increase. Men with at least one period of income increase have an 81 percent higher risk of ending up in poor SRH compared to the reference group. The estimates for women in the same category, however, reveal the opposite association, although the results are non-significant (OR 0.78, CI 95% 0.55-1.12). In the mixed category of “inconsistent” income, we detect an increase in odds of poor health for men by 61 percent and a non-significant increase for women by 32 percent.

### Model 4: Relative changes in absolute income

Model 4 shows the changes in income based on absolute income, i.e. the nominal amount of income a person received. Income loss in combination with stable income reveals the same estimate for men as shown in Model 3 (OR 2.02, CI 95% 1.22-3.33). Increased odds ratios for poor health are also found for women (OR 1.50, CI 95% 0.99-2.28). The estimates for income increase are slightly below the reference category, but non-significant. In contrast to Model 3, inconsistent income does not reveal a significant association with poor SRH for men, but a notable though non-significant estimate for women.

### Model 5: Regression slopes of absolute income over time

The results based on the individual income slopes are shown in Model 5. The estimates somewhat contradict the findings from Model 3 and 4. The analysis of the male sample demonstrates a significant gradient between income trends and health, although the single estimates remain non-significant. The estimates shown for women suggest a U-shaped association between income trends and health. Increases in income are significantly associated with better health. In addition, Model 5 shows the linear gradient for the association of income slopes and SRH. When collapsing the five categories into a single estimate, a significant association between the steepness of the income trajectories for men and women is observed.

## Discussion

The aim of this study was to explore income changes and their relevance for subsequent self-rated health, by implementing income histories based on register records into a cross-sectional survey. The type of analysis we presented in this study was restricted by a cross-sectional design, with variables accounting for various types of income development over time and length in a specific income position. The cross-sectional nature of the used data and the characteristics of the variables determined the choice of analysis. We attempted to circumvent the static nature of cross-sectional analysis by including income histories that were recoded into categorical variables applicable for logistic regression. Generally, cross-sectional analysis underestimates the flexible nature of income and conceals that the aspect of time within the association between income and health might be potentially important [6].

### Implications of enduring low income position

The first model revealed the lagging effect of prior low income which seems to determine the health outcome. Using an alternative cut-off point for the level of deprivation and cruder time intervals, Benzeval and Judge [6] identified an increased prevalence of poor health after one and three years in low income position, which is somewhat similar to our findings. The same study found lagged effects of income on health where income was associated with SRH and other health measures up to five years back in time. Although we could not detect a gradient in the lag time of low income position, the delayed effect of income deprivation is noteworthy. A corresponding lag time gradient between socio-economic position and health has been shown in previous studies, suggesting that the accumulation of low occupational position determines health later in life [15, 16]. Kaplan et al. [17] could show that benefits of socio-economic (dis)advantage accumulate for psychological well-being over time. We therefore presume that time is an important aspect in the income-health relationship. In general, the findings from previous research seem to support the relevance of short-term effects on health, whereas long-term consequences as shown by Blakely et al. [10] increase the possibility of health selection [9].

### Implications of income change

In a set of models, we accounted for short-term income change and income mobility within a six-year period and the subsequent outcomes for SRH. The modeling strategy distinguished between changes in absolute income and changes in the relative rank position in the income distribution, which led partially to fairly different results. Both approaches revealed that a substantial loss of income at one or more occasions during the past six income years is associated with an increased risk of poor health (more for men than for women). Inconsistent rank positions in the income distribution yielded increased estimates of poor SRH for men, whereas inconsistent absolute income disclosed a higher prevalence of poor SRH only among women. Counter-intuitive and somewhat paradoxical were the health implications of upward income for men when change in relative rank position was accounted for. The somewhat inconclusive findings resemble a previous study that explored changes in income over time by using an earlier wave of the same data: Lundberg and Fritzell [18] found an association between income change and health with more plausible associations for men than for women.

It is widely accepted that low income is risk factor for poor health. Evidence in previous research is mostly based on income inequality measured closest to health outcomes, but it is uncertain whether income affects only influence health instantaneously [19]. The presented findings based on negative income trends somewhat support the notion that income also has longer-term health implications. Therefore, downward income trends and inconsistent income histories with temporal periods of low income may have similar negative health implications, as currently demonstrated for low-income position. In addition, inconsistent income positions over time likely discloses unsteady living conditions and socio-economic disadvantages imposing lower consumption ability and psycho-social stress that influence health adversely [4, 5, 11]. Unstable (i.e. inconsistent) income may also be regarded as an indicator of temporal status inconsistency that in itself has been identified as determinant for health. The adverse health effects of inconsistent social position have been demonstrated earlier [20, 21]. Whereas the findings for income decrease are intuitive and straightforward, the health outcomes for income increase among men (and sex differences in inconsistent income position) were puzzling and somewhat unexpected. Health hazards stemming from increasing income may be explained with changes in consumption pattern and health behavior. Also comparison mechanisms such as relative deprivation may have contributed to the findings, reflecting that people fall behind comparable others and their own expectations with regard to income [22–24].

The study uses one income variable; however it was scaled in different ways throughout the presented analysis. The contrasting findings between absolute income and rank position indicate that the type of scaling used on the income variable is important with respect to the health outcome. For example, previous studies on the causal implications of income on health have shown that the method of scaling income may lead to different conclusions regarding health [25]. The income variable modeled as absolute income draws on material conditions of the individual. Whereas rank positons in income distribution acknowledge the role of social comparisons with significant others, and thus emphasize the psycho-social dimension of income inequality [26, 27]. In practice, however, both types of scaling widely overlap which makes it cumbersome distinguishing between the absolute and relative aspects of income position [28].

The overall income development in the study population may have influenced the presented results. In particular the findings based on absolute income follow the trends of the overall income development on the population-level. The findings based on relative rank measures, however, are less prone to fluctuations in income on the national level and are more influenced by changes in the income distribution. The compression of the income distribution affects the relative distances between lower and higher income groups and thus the rank positions in the income distribution [3, 29].

Not to be downplayed, the rise and fall of individual income over time is primarily a consequence of preceding changes in the individual’s employment and labor market position. In the presented analysis, confounding from employment status may have occurred and affected the findings because an appropriate control variable was not available in the data material. Previous studies adjusting the income-health relationship for employment showed that magnitudes in poor health become attenuated [30]. However, it has also been reasoned that the adjustment is problematic as employment status can be regarded as a proxy for health. Poor health status is a major cause for interrupted work biographies and non-active labor force [31]. In the same study it was further argued that employment and income belong to distinct causal pathways, with income accounting for material living conditions and employment capturing aspects of health behavior. We therefore regard the omission of employment status as tolerable.

### Limitations

The data format and structure restricted the possibilities of data analysis in this study. The presented analyses proposed to enhance cross-sectional studies, but are nevertheless not powerful as true longitudinal time-series approaches. A shortcoming in the current study is the availability of the outcome measure at only one time-point during the observation period. For the same reason, an appropriate adjustment for socio-economic positions was impeded and relied on education alone. Thus, the temporal ordering of social position and health status cannot properly be determined. At least some of the findings may therefore be a result of reversed causality with health influencing income [32].

The inconsistent and not always conclusive findings for women in this study may because women’s income depends to some extent on that of their partner’s socio-economic situation [25, 33]. Also, the type of income measure might have influenced the outcomes. Since information on household composition could not be linked to the specific years when income was received, the analysis relied on individual disposable income. Using household income adjusted for household composition would certainly produce different results and likely provide a more accurate account of women’s conditions and their income-health associations.

Income information may be biased due to an overall increase in income. However, as inflation in Sweden was comparably low during the observation period (0.5 percent annually), its influence on the used income variables and results is presumed to be rather low.

The lack of longitudinal information on control variables impeded investigation into the specific reasons for changes income. It is very likely that changes in income coincide with events like unemployment, family dissolution etc. which themselves explain variations in SRH.

## Conclusions

Although we were able to show that the income distribution is relatively stable over time and that income inequality did not change substantially during the considered period, individual income mobility did occur. Following individual income trajectories, we showed that individual income change is an important determinant for health. This holds true independent of level of income. Although we used relatively old data, covering the 1990s in Sweden, we still believe the results are relevant. Income inequality in Sweden has increased in the past decade, and has certainly affected the income-health relationship.

The study showed that the dimension of time and thus the lagged effects of income should be considered when studying income inequality in health. In absence of reference data, the analytical design could not control for potential influences stemming from welfare reforms and retrenchments in the wake of the 1990’s recession in Sweden. However, it was shown that loss of income is likely a threat for health while increases income revealed rather inconsistent results.

Although this study could not identify a consistent pattern and clear direction of associations between income and self-rated health over time, it was shown that the time dimension matters. In particular income instability – in either upward or downward trends of income – show an adverse association with health.
